# Toxicological Responses of Juvenile Gilthead Seabream to Enniatin B and Fumonisin B1

**DOI:** 10.3390/ijms26125676

**Published:** 2025-06-13

**Authors:** Flávia V. Mello, Cheila Pereira, Busenur Özkan, Ana Luísa Maulvault, Florbela Soares, Pedro Pousão-Ferreira, José O. Fernandes, Sara C. Cunha, António Marques, Patrícia Anacleto

**Affiliations:** 1 MARE—Marine and Environmental Sciences Centre & ARNET, Aquatic Research Network, Faculdade de Ciências da Universidade de Lisboa (FCUL), Campo Grande, 1749-016 Lisboa, Portugal; 2 LAQV-REQUIMTE, Laboratory of Bromatology and Hidrology, Faculty of Pharmacy, University of Porto, Rua Jorge de Viterbo Ferreira 228, 4050-313 Porto, Portugal; 3 Division of Aquaculture, Upgrading and Bioprospection, IPMA, I.P.—Portuguese Institute for the Sea and Atmosphere, Av. Doutor Alfredo Magalhães Ramalho 6, 1495-165 Lisboa, Portugal; 4 UCIBIO—Applied Molecular Biosciences Unit, NOVA School of Science and Technology, NOVA University of Lisbon, Quinta da Torre, 2819-516 Caparica, Portugal; 5 Associate Laboratory i4HB Institute for Health and Bioeconomy, NOVA School of Science and Technology, NOVA University of Lisbon, Quinta da Torre, 2829-516 Caparica, Portugal; 6 Aquaculture Research Station of Olhão (EPPO), IPMA, I.P.—Portuguese Institute for the Sea and Atmosphere, Av. do Parque Natural da Ria Formosa S/N, 8700-194 Olhão, Portugal; 7 CIIMAR—Interdisciplinary Centre of Marine and Environmental Research, University of Porto, Terminal de Cruzeiros do Porto de Leixões, Av. General Norton de Matos S/N, 4450-208 Matosinhos, Portugal

**Keywords:** mycotoxins, biomarkers, fish feed, fish, toxicology

## Abstract

The replacement of ingredients from animal sources with plant-based ingredients is increasing the risk of contamination by mycotoxins in aquafeeds, potentially causing detrimental effects on fish welfare. However, limited research has been carried out so far on the impact of mycotoxins on fish health. Hence, the aim of this study was to assess the toxicological effects of the dietary emerging (enniatin B, ENNB) and regulated (fumonisin B1, FB1) mycotoxins (150 µg/kg) in different tissues of juvenile gilthead seabream (*Sparus aurata*) after 28 days of dietary exposure. Fitness indexes, plasma metabolites, and biomarkers of oxidative stress, metabolism, cellular, and neurotoxic damage were assessed. The exposure to each mycotoxin was sufficient to cause distinct effects in fish tissues. ENNB appears to be the most harmful mycotoxin to *S. aurata*, inducing changes on alkaline phosphatase and lipase activities in plasma, as well as protein and lipid degradation in liver. Increased lipid degradation was also induced in the brain by FB1 alone or combined with ENNB, whereas the exposure to the mixture inhibited acetylcholinesterase activity. Overall, this study contributes by highlighting the toxicological attributes of ENNB, thus reinforcing the need to include this mycotoxin in future legislation.

## 1. Introduction

Aquaculture is considered a sustainable solution for the growing global food demand, being responsible for 17% of animal protein consumed worldwide [1]. With aquatic animal production expected to increase by 15% until 2030, sustainable and equitable aquaculture development strategies are essential to preserve aquatic ecosystem health, animal welfare, pollution levels, and biodiversity [2].

In the last decades, the use of fish meal and fish oil has been reduced and gradually replaced by greener alternatives, such as plant-based ingredients (e.g., cereals), in order to improve the profitability and sustainability of the sector [3]. However, such alternatives raise safety and toxicity concerns due to mycotoxin contamination, mainly if conditions during crop growing, processing, or storage are favorable to mycotoxigenic fungal growth [4]. Currently, the prevalence for the detected mycotoxins in global food crops can be up to 60–80% [5]. Several studies have reported mycotoxin contamination in feed ingredients, aquafeeds and/or tissues of European farmed fish species [6,7,8]. High levels of individual mycotoxins (around 5 mg/kg) have been found in aquafeed ingredients [6,9]. Among the most frequently detected mycotoxins are the enniatins (ENNs) and fumonisins, whose presence is increasingly reported in multi-mycotoxin studies. Among the ENNs, enniatin B (ENNB) has been found in 90% of all tested European marine aquafeeds and 65% of farmed fish fillets, such as gilthead seabream (*Sparus aurata*) and European seabass (*Dicentrarchus labrax*) [10,11,12,13]. This mycotoxin can cause stunted growth, but unrelated to feed utilization capacity, and anemia, and also can trigger an acute inflammatory response in the intestine [14,15]. However, little is known about sub-chronic ENNB effects in in vivo trials with farmed fish species [12,14,16,17].

In addition to ENNs, the legislated FB1 is the most predominant fumonisin, being classified in Group 2B as a possibly human carcinogen according to the International Agency for Research in Cancer (IARC). In addition, it shows systemic toxicity, including neurotoxicity, hepatotoxicity, nephrotoxicity and mammalian cytotoxicity [18]. Moreover, FB1 inhibits the biosynthesis of sphingolipids, compromising the membrane characteristics, and altering cellular functions [19]. Additionally, fumonisins generally cause a fish weight gain reduction, a decrease in hematocrit, erythrocytes, hemoglobin, and protein constituents [20]. Nevertheless, only about 3% of studies on mycotoxins have used fish as an in vivo animal model [21].

Based on the public health risks that these mycotoxins can pose, the European Union has set maximum permitted levels for some mycotoxins (Commission Regulation (EC) No 2023/915 and EC No 401/2006; Commission Recommendation (EU) 2016/1319), including total FBs (FB1 and FB2) in feedstuffs [22,23,24]. On the other hand, emerging mycotoxins, including ENNB, have not yet been regulated and maximum levels have not been established in feeds and food products [25]. Their high incidence and possible health effects have, thus, recently prompted a growing interest among the scientific community and policy-makers in evaluating the mycotoxins prevalence in food and feeds, as well as their potential toxicity in animals and humans [26].

Like many other contaminants, mycotoxins are also responsible for reactive oxygen species (ROS) production, which have detrimental effects on cellular molecules and structures, causing lipid peroxidation, protein oxidation, DNA damage, and the imbalance of the intracellular redox status [27]. This occurs when the antioxidant defense system fails to rapidly counterbalance ROS production [28]. Changes in the activity of enzymes are physiological responses that have been used for decades as biomarkers of fish health deterioration and evidence of stress-related adverse biological effects [29]. Some biomarkers help in understanding the effectiveness of antioxidant defenses in limiting ROS-mediated damage to intracellular macromolecules (DNA, proteins, and lipid) or cell death [30]. Thus, oxidative stress and the effects of ROS produced by mycotoxins can be assessed by evaluation of the cellular antioxidant defense systems, metabolic biomarkers, and indicators of cellular and neurotoxic damage [31]. In addition, changes in plasma biochemical parameters are a useful tool to evaluate physiological disturbances occurring in the animal [32]. For example, increased levels of liver enzymes, alanine aminotransferase, and aspartate aminotransferase, in farmed Atlantic salmon plasma exposed to high levels of ENNB (83 mg/kg feed) indicated liver injury, and therefore, liver toxicity [14].

Thus, this work aimed to address the present literature gap on in vivo mycotoxins exposure in farmed fish by assessing the toxicological responses (metabolic and antioxidant activity, heat shock response, protein degradation, and neurotoxicity) in tissues (muscle, liver, gills, and brain) of juvenile *Sparus aurata* exposed to ENNB and FB1. This species was selected as a biological model because it is a commercially and ecologically important fish species in aquaculture, being widely cultured in the Mediterranean area [1] and since it is a carnivorous predatory species, it is a suitable representative model to assess toxicological impacts at multiple biological levels. Moreover, the two mycotoxins chosen are among the most common and most problematic mycotoxins found in cereals and other feed ingredients used in the aquafeeds production for farmed fish [7].

## 2. Results and Discussion

### 2.1. Fitness and Growth Performance

Morphometric measures, and the fitness and growth performance of juvenile *S. aurata* exposed to four treatments at day 0 (T0) and day 28 (T28) are shown in Table 1. Fish exposed to FB1 (90.3 ± 0.6 g) or to the mixture of both mycotoxins (FB1 + ENNB; 87.8 ± 5.1 g) had a significantly higher weight (*p <* 0.05) than those exposed only to CTRL and ENNB. However, fish exposed only to FB1 showed a higher body length (17.8 ± 0.5 cm; *p <* 0.05) than fish from the other treatments. However, a significantly reduced relative growth rate (RGR) in fish exposed to ENNB (13.4 ± 3.2%) was observed in comparison to the other three treatments (24.3–29.0%). Therefore, ENNB alone seems to have a moderate effect size (η^2^ = 0.30) on the RGR of this species (F(3, 7) = 11.954, *p* < 0.01). Berntssen et al. found a stunted growth in other farmed fish (Atlantic salmon, *S. salar*) exposed to higher concentrations of dietary ENNB (5.2 and 83 mg/kg) and for a more prolonged time (37 to 69 days) [14]. Moreover, no mortality was observed in all treatments, confirming that the exposure time of the trial, as well as the mycotoxin level used, were not lethal to juvenile gilthead seabream.

In this study, the exposure to mycotoxins FB1 and ENNB did not affect the daily feed intake (FI) (*p >* 0.05), which ranged from 1.06 to 1.14 g/fish/day. However, the feed conversion rate (FCR) significantly increased in fish exposed to ENNB (*p <* 0.05), indicating that the consumed feed is less well converted into weight in this treatment (2.74 g feed intake/g weight gain; Table 1). The FCR in fish from the control and other treatments are in accordance with those observed for gilthead seabream in other studies [33,34]. In contrast, Berntssen et al. did not observe effects of ENNB on the FCR of exposed salmons (1.0–1.2 feed intake/g weight gain), while fish fed beauvericin (BEA), an emerging mycotoxin, at medium and high levels (4.8 and 46 mg/kg), had an increased FCR (~60%) [14]. Similarly, in vitro studies showed ENNB effects such as mitochondrial disruption with impaired energy formation [35] and acute intestinal inflammation in Atlantic salmon [15], and cytotoxic effects on cell lines (Caco-2) in an intestinal environment [36].

Although VSI was not significantly affected by mycotoxins (*p >* 0.05), fish exposed to FB1 had a lower HSI (*p* < 0.05). Similarly, in a study performed with rainbow trout (*Oncorhynchus mykiss*) exposed during a 6-week restrictive and 2-week *ad libitum* feeding periods, HSI was affected by a fumonisins mixture (FB1, FB2, and FB3) [37]. In fact, some authors reported that FB1 is toxic to the liver in several species, inducing metabolism changes that can interfere with lipid and glycogen deposition due to stress conditions [38]. Other studies have shown more severe FB1 effects on the metabolism of sphingolipids, which participate in diverse cell processes, mediating cytotoxicity and apoptosis [18]. The obtained Fulton’s condition index (K) indicated that *S. aurata* juveniles were in a good health condition [39] since K was higher than 1 in all treatments (1.5–1.7; Table 1), suggesting greater energy reserves, mainly lipids [40], contributing to improved health, reproductive fitness, and growth, among other factors [41]. Curiously, fish exposed to combined mycotoxins (FB1 + ENNB; 1.7 ± 0.1) showed a slightly higher condition’s factor than the CTRL (1.5 ± 0.1).

### 2.2. Plasma Biochemical Profile

In general, FB1, whether alone or combined with ENNB, did not affect the plasma constituents of exposed fish, suggesting no disturbance in the species’ plasma (Table 2). However, ENNB alone showed alterations in two biochemical analytes (Table 2), i.e., a significant reduction in alkaline phosphatase activity (ALP; 229 ± 40 U/L; *p* < 0.05) and an enhanced lipase activity (LIPA; 36.5 ± 9.7 U/L; *p <* 0.05) in comparison with the CTRL treatment (339 ± 84 and 24.4 ± 3.3 U/L, respectively). The increase of lipase activity in the ENNB treatment suggests a higher lipid mobilization in the liver and adipose tissues in order to supply fatty acids to peripheral tissues [42]. However, the unchanged HSI showed that this mobilization from the liver seems not to be significant in this treatment. Probably, the use of the lipid reserves is promoted as a consequence of ENNB effects on metabolism or energy expenditure, as shown by the lower growth (RGR) and higher FCR of ENNB-exposed fish (Table 1).

Concerning ALP activity, the decline observed in ENNB-exposed fish can be attributed to a decrease in enzyme synthesis and turnover rates, potentially due to lower metabolic demands [43]. ALP is a non-specific membrane-bound enzyme involved in detoxification and is commonly considered an indicator of tissue damage resulting from toxic exposure or other stress conditions [44]. Its presence in plasma is generally associated with a release from damaged cells, such as those in the liver, bone, intestine and kidney, into the extracellular fluid [45]. Although reduced ALP levels are less frequently reported, several pesticides (e.g., cypermethrin, chlorpyrifos, simazine, and NeemAzal), have also been shown to reduce ALP activity in fish plasma [46,47,48]. For example, Bowser et al. observed a time-dependent decrease in ALP activity in the plasma of Atlantic salmon exposed to elevated nitrite concentrations (25 mg/L) over 12 days [49], which was associated with damage to red blood cells, severe anemia, and reduced ALP biosynthesis in hepatic and intestinal cells [50]. While the exact mechanisms underlying ALP reduction are not fully understood, some studies suggest that alterations in membrane integrity, the dysfunction of ion channels, and an impaired absorption of essential ions (e.g., zinc, magnesium, and phosphorus) may contribute to a decreased ALP activity [48]. These processes could also interfere with calcium absorption and the dephosphorylation pathway in osteocytes, where ALP plays a key role in mineralization [48]. The same pattern for lipase and ALP was also observed in gilthead seabream after one week of starvation [32]. Therefore, these plasma biochemical parameters suggest that ENNB affects the cell membrane, and consequently, the feed and/or energy utilization efficiency of this species since FCR was also higher in this treatment.

### 2.3. Toxicological Responses

#### 2.3.1. Oxidative Stress Biomarkers

**Figure 1:**
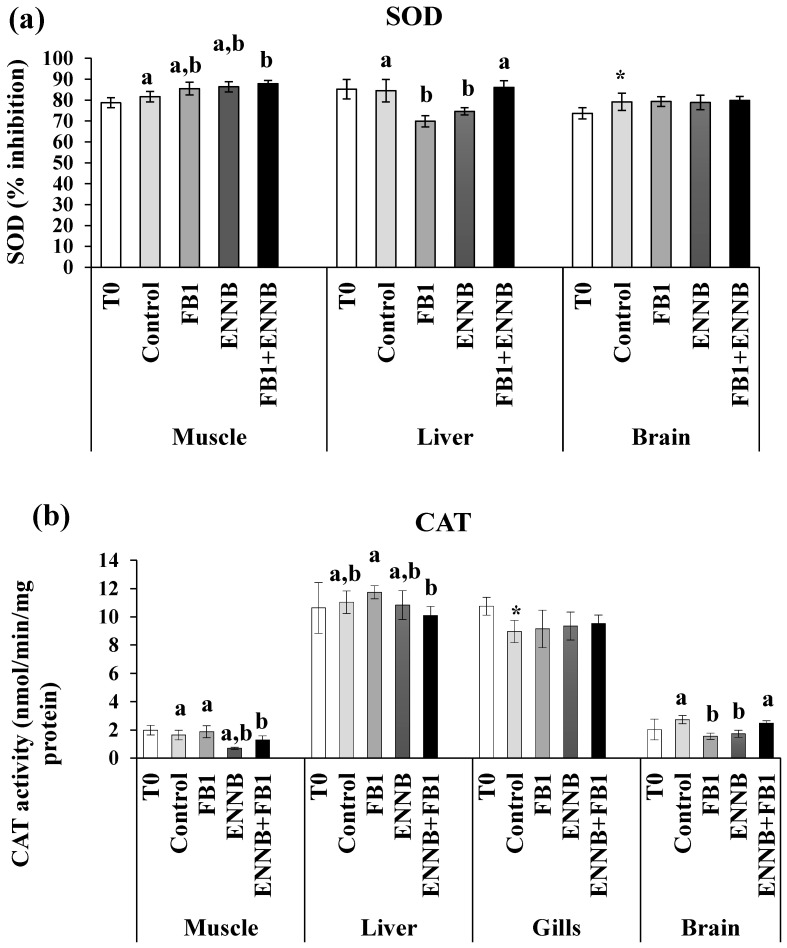

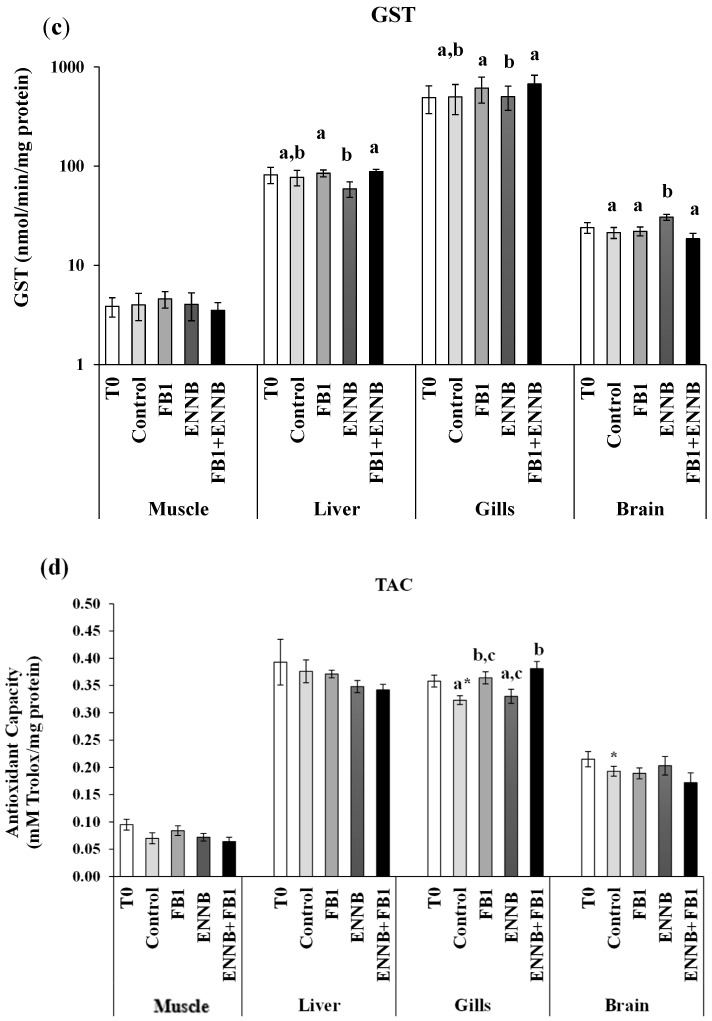
Antioxidant enzymes activity. (**a**) SOD, % inhibition; (**b**) CAT, nmol/min/mg protein; (**c**) GST, nmol/min/mg protein; (**d**) TAC, mM Trolox/mg protein) in the muscle, liver, gills, and brain of juvenile *S. aurata* (average ± standard deviation; *n* = 6) before (T0) and after 28 days of exposure to the different experimental aquafeeds. Different letters (a–c) indicate significant differences between the treatments at day 28 (ANOVA or Kruskal–Wallis; *p* < 0.05). Asterisks (*) indicate significant differences between T0 and CTRL (*t* test or Mann–Whitney; *p* < 0.05). Abbreviations: T0—initial sampling point (at day 0); CTRL—control; FB1—fumonisin B1; ENNB—enniatin B.

Antioxidant enzyme activity (superoxide dismutase—SOD, glutathione S-transferase GST and catalase—CAT) and the total antioxidant capacity (TAC) in fish tissues (muscle, liver, gills, and brain) are shown in Figure 1. The present results showed that mycotoxin exposure, either alone or combined, affected the antioxidant machinery in a tissue- and biomarker-specific way. A significant increase of the inhibition percentage of SOD activity in the *S. aurata* muscle (*p* < 0.05) was caused by ENNB combined with FB1 (FB1 + ENNB) in comparison with the CTRL. On the other hand, SOD activity was significantly enhanced in the liver of fish exposed to FB1 (*p* < 0.0001) and ENNB alone (*p* < 0.05) (Figure 1a). These metalloenzymes comprise the very first step of defense against reactive oxygen species in biological systems, in which superoxide radicals are converted into oxygen peroxide (H_2_O_2_) [51]. Although the alone mycotoxins seem to stimulate the antioxidant machinery, combined mycotoxins can be related to an excessive production of superoxide anion during the detoxification process and consequent SOD deactivation and/or H_2_O_2_ accumulation [52]. Furthermore, the exhaustion of the cell’s capability in compensatory responses to severity of stressors and duration overwhelm can occur [53], and consequently, the organisms become more susceptible to stress and to potential adverse effects.

With regard to CAT, the exposure to both mycotoxins resulted in higher CAT activities in the liver (9.9–11.8 U/mg protein, *p <* 0.05) and gills (8.3–10.7 U/mg protein, *p <* 0.001) tissues. Compared to the CTRL, ENNB seems to lead to significant decreases in CAT activity in the muscle (0.700 ± 0.079; *p* < 0.01) and brain (1.724 ± 0.251; *p* < 0.05) (Figure 1b). Additionally, FB1 also affected CAT activity in the brain (1.550 ± 0.221; *p* < 0.01). Since CAT is responsible for the conversion of H_2_O_2_ into water (H_2_O) and molecular oxygen (O_2_) during the detoxification phase, its inhibition can be caused by the excessive production of H_2_O_2_ which may be harmful to the tissues, leading to an increase in oxidative stress [38]. In this way, the antioxidant machinery was unable to compensate for an excessive substrate production in these tissues when exposed to FB1 or ENNB. Similarly, Maulvault et al. described CAT inhibition in the muscle and gills of juvenile meagre (*Argyrosomus regius*) exposed to venlafaxine (a pharmaceutical active compound) via feed (160 µg/kg dw) for 28 days [38]. Also, in another study conducted with European seabass (*D. labrax*), a significant decrease in CAT activity in fish muscle was observed when exposed to diclofenac (a pharmaceutical drug) via feed (500 ng/kg dw) for 28 days [54].

Concerning GST, its activities were not changed by the mycotoxin-contaminated aquafeeds in muscle, liver, and gills (*p* > 0.05) when compared to the CTRL treatment (Figure 1c). In contrast, an opposite tendency occurred in the fish brain, where ENNB alone caused the enhancement of GST activity (27.2%), and the same tendency was also observed by Maulvault et al. in *D. labrax* [54]. Considering that GST is involved in the detoxifying of various harmful compounds by conjugating them with glutathione [30], its enhancement contributes to the detoxification of harmful ROS and their reaction products and prevents the occurrence of oxidative stress and cellular damage in the tissue [55].

In relation to TAC, the potential of body fluids to neutralize oxidants was enhanced only in fish gills exposed to FB1, either alone or in combination with ENNB (FB1 or FB1 + ENNB treatments, respectively; Figure 1d). This increase in TAC levels may represent a physiological response to counteract the elevated production of ROS induced by FB1. Furthermore, given the high metabolic rate and continuous exposure of gill tissue to the external environment, gills are particularly susceptible to oxidative damage [56]. The observed enhancement of TAC in the gills may thus represent a local compensatory antioxidant response to maintain redox homeostasis under FB1-induced oxidative stress. Similar increases in antioxidant defense mechanisms have been reported in fish gills exposed to other environmental stressors and contaminants (e.g., [57]), suggesting that localized antioxidant responses may be triggered in tissues directly affected by xenobiotics.

The present findings showed that the highest antioxidant defense system was generally observed in the liver and gills of gilthead seabream, which highlights their importance in scavenging ROS produced by oxidative stress [58]. Liver is the primary organ involved in the metabolism of xenobiotics and therefore in the detoxification process. Although no marked pattern has been observed in each tissue or contaminated treatment, effects on the antioxidant machinery of this species were identified as a way to prevent oxidative damage.

#### 2.3.2. Cellular Damage

##### Lipid Peroxidation

**Figure 2:**
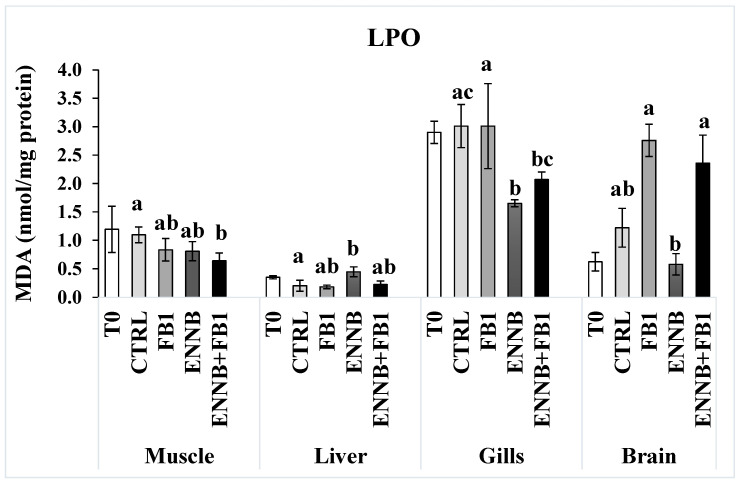
Lipid peroxidation (LPO) expressed by MDA concentration (nmol/mg protein) in the muscle, liver, gills, and brain of juvenile *S. aurata* (average ± standard deviation; *n* = 6) before (T0) and after 28 days of exposure to the different experimental aquafeeds. Different letters (a–c) indicate significant differences between the treatments at day 28 (ANOVA or Kruskal–Wallis; *p* < 0.05). Abbreviations: T0—initial sampling point (at day 0); CTRL—control; FB1—fumonisin B1; ENNB—enniatin B.

LPO measures the lipid degradation by the attack of oxidants (free radicals or non-radical species) to lipids that contain carbon–carbon double bond(s), especially polyunsaturated fatty acids (PUFAs) but also glycolipids, phospholipids, and cholesterol [59]. A significantly enhanced LPO was observed in the liver of fish exposed to ENNB alone (122%; Figure 2), which revealed cellular damage induced by ENNB. In fact, substantial permeability changes induced by ENNs on cell membranes may contribute to lipid peroxidation as mentioned by Catalá et al. [60]. Thus, the antioxidant response offered protection from oxidative stress to some extent, not being totally efficient particularly in the liver, which is the main organ of the metabolization of toxins. In contrast, LPO significantly decreased in mycotoxins mixture-exposed fish muscle (FB1 + ENNB; 42%) and ENNB-exposed fish gills (45%). This can probably be due to the alterations in the rate of unsaturation and fatty acid chain length found after mycotoxin exposure that can lead to a decrease in LPO levels as reported by some studies (e.g., [61]). As shown in Figure 2, SOD was activated precisely in the muscle of fish exposed to the mycotoxin’s mixture. Therefore, PUFAs, such as membrane phospholipids, seem to be protected in gills and muscle, avoiding damage to membrane structure and its functions [28,62]. Additionally, no significant differences were observed between sampling times, i.e., T0 and T28 in any tissue, whereby no influence of time occurred on the LPO (*p >* 0.05).

##### Protein Chaperoning and Degradation

**Figure 3:**
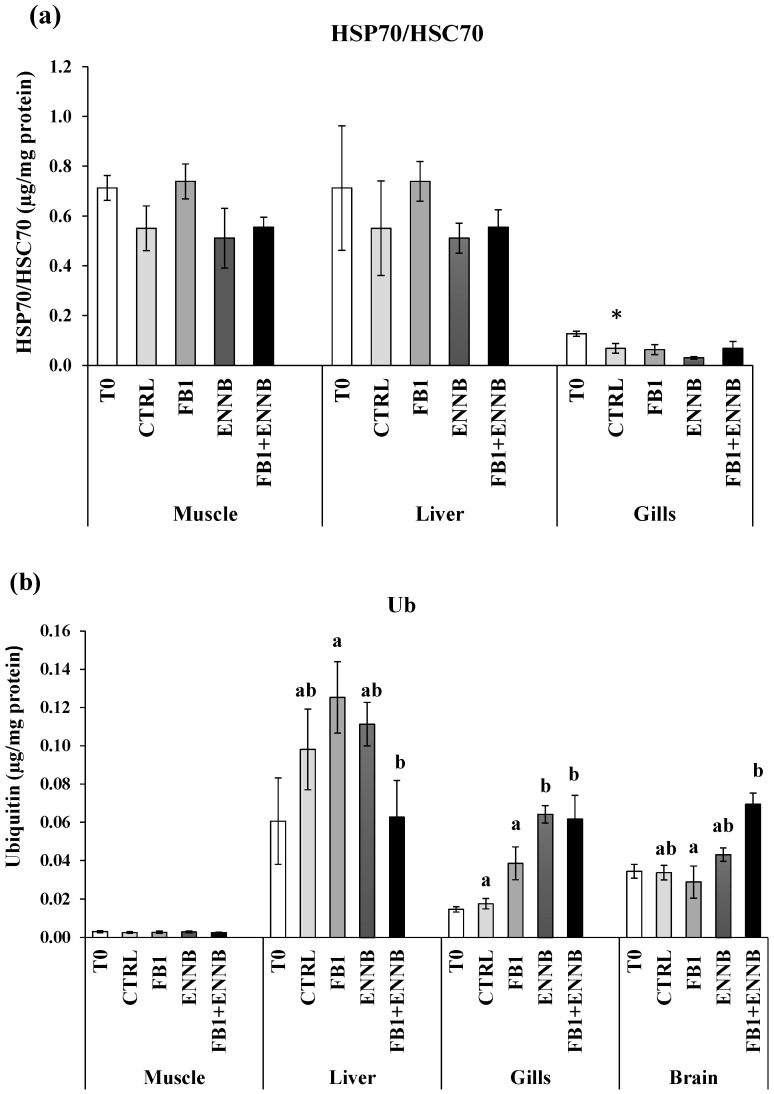
(**a**) Heat shock proteins (HSP70/HSC70; µg/mg protein) and (**b**) total ubiquitin concentration (Ub; µg/mg protein) in the muscle, liver, gills, and brain of juvenile *S. aurata* (average ± standard deviation; *n* = 6) before (T0) and after 28 days of exposure to the different experimental aquafeeds. Different letters (a,b) indicate significant differences between the treatments at day 28 (ANOVA or Kruskal–Wallis; *p* < 0.05). Asterisks (*) indicate significant differences between T0 and CTRL (*t* test or Mann–Whitney; *p* < 0.05). Abbreviations: T0—initial sampling point (at day 0); CTRL—control; FB1—fumonisin B1; ENNB—enniatin B.

Heat shock proteins (HSP70/HSC70) are molecular chaperones involved in the folding of unfolded and misfolded polypeptides that were damaged by cell stress. This prevents the formation of irreversible protein aggregates, reactivates inactive proteins, disassembles proteins from aggregates, and degrades proteins together with proteases [63]. A significantly higher HSP70/HSC70 concentration was found in gilthead seabream muscle and liver (0.51–0.74 µg/mg protein) when compared to gills at T0 (0.03–0.13 µg/mg protein; Figure 3a). However, there were no changes in HSPs production in the muscle and liver tissues after an exposure period of 28 days to mycotoxins. Over time, significant differences were only found in gills (*p* < 0.05), where a decreased HSPs production was observed in the CTRL after a 28 day-exposure in comparison to the T0 (Figure 3a). The maintenance of HSPs concentration indicates that proteins in these tissues were not damaged by the presence of the mycotoxins in feed, which did not induce HSPs synthesis or an impaired cellular response.

When irreversible protein anomalies occur, ubiquitin production is also initiated to signal and eliminate such proteins [64]. Ubiquitins are also involved in immune responses, transport through membranes, DNA repair, and chromatin remodeling [65]. Liver tissue presented a higher baseline of ubiquitins in fish not exposed to mycotoxins (T0–0.06 ng/mL/mg protein and CTRL—0.1 ng/mL/mg protein; Figure 3b). Considering all dietary treatments, gilthead seabream muscle showed the lowest Ub production (0.025–0.030 µg/mg protein). Although no significant changes were found after 28 days of exposure in the liver in comparison to the CTRL, the Ub concentration was significantly higher in the liver exposed to FB1 alone than in the mixture with ENNB (FB1 + ENNB; *p* < 0.05). Contrarily, significantly higher Ub levels were observed in the brain exposed to the mixture than FB1 alone (*p* < 0.05). Nevertheless, a significantly enhanced Ub production was observed in ENNB alone (265%) or in mixture with FB1 (FB1 + ENNB) in fish gills (250%), indicating irreversibly damaged protein. Since the ubiquitin production was increased and HSP70 was unchanged, it confirms the impairment of heat shock response in gilthead seabream exposed to ENNB alone or combined with FB1. This impairment may occur when organisms experience severe or prolonged stress conditions, which can lead to a marked reduction in protein synthesis due to its high energetic process [54].

##### Neurotoxicity

**Figure 4:**
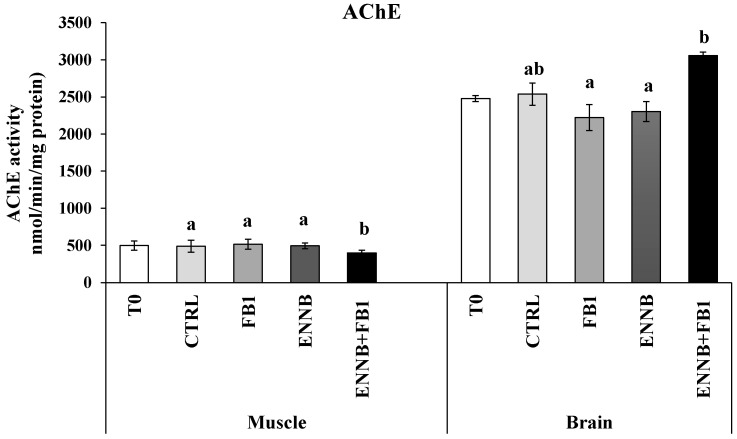
Acetylcholinesterase (AChE) activity (nmol/min/.mg protein) in the muscle and brain of juvenile *S. aurata* (average ± standard deviation; *n* = 6) before (T0) and after 28 days of exposure to the different experimental aquafeeds. Different letters (a,b) indicate significant differences between the treatments at day 28 (ANOVA or Kruskal–Wallis; *p* < 0.05). Abbreviations: T0—initial sampling point (at day 0); CTRL—control; FB1—fumonisin B1; ENNB—enniatin B.

The enzyme acetylcholinesterase (AChE) is responsible for terminating the transmission of nerve impulses at cholinergic synapses through the hydrolysis of the neurotransmitter acetylcholine [66]. Previous studies have reported the inhibition of AChE activity in aquatic organisms also exposed to other contaminants, such as flame retardants, pesticides, and pharmaceuticals [67,68,69]. The AChE activity is generally higher in the brain, although it is also very active in skeletal muscle [70], which was observed in our study (brain: 2221–3054 nmol/min/mg protein; muscle: 400–514 nmol/min/mg protein). In this study, no changes were observed in the contaminated *S. aurata* brain compared to the CTRL (*p* > 0.05), but an inhibition of AChE activity was found in mycotoxins mixture-exposed fish muscle (ENNB + FB1; 18.2%; *p* < 0.05; Figure 4). These mycotoxins, when combined at this concentration in aquafeed, seem to be neurotoxic in muscle cells since the neurotransmitter accumulates with decreased AChE activity, resulting in deleterious effects for animals. Although no remarkable changes were observed in the behavior of the fish in this study, in other studies they were detected, which can be probably due to the muscle contraction that is affected since acetylcholine is important for muscle functioning [68]. Moreover, as the activity of AChE is higher in motor neurons than in sensory neurons, this effect manifests markedly in muscle. FB1, in turn, has known neurotoxic effects, such as neurological dysfunction and the reduction of cell body areas in neuronal populations [18]. Although it is difficult to cross the blood–brain barrier, damage in more sensitive cells and the excitability of neurons and neural networks can lead to neurological abnormalities [71]. Nonetheless, the neurotoxic effect of the combination of both mycotoxins by chronic exposure is still unknown and our results suggest this potential in the studied conditions.

#### 2.3.3. Metabolic Responses

**Figure 5:**
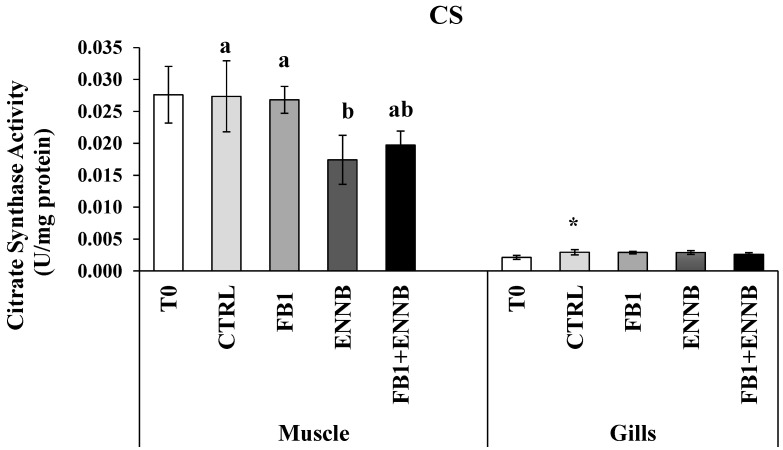
Citrate synthase activity (U/mg protein) in the muscle and gills of juvenile *S. aurata* (average ± standard deviation; *n* = 6) before (T0) and after 28 days of exposure to the different experimental aquafeeds. Different letters (a,b) indicate significant differences between the treatments at day 28 (ANOVA or Kruskal–Wallis; *p* < 0.05). Asterisks (*) indicate significant differences between T0 and CTRL (*t* test or Mann–Whitney; *p* < 0.05). Abbreviations: T0—initial sampling point (at day 0); CTRL—control; FB1—fumonisin B1; ENNB—enniatin B.

Citrate synthase (CS) is a citric acid enzyme indicator of the overall aerobic metabolic potential in the mitochondria [72]. Changes in the metabolic machinery can lead to a shift in the energy production mode by the Krebs cycle and reflect on the aerobic scope and performance of organisms [73]. Basal CS levels observed in gilthead seabream muscle (0.024 ± 0.005 U/mg protein) were higher than in gills (0.003 ± 0.000 U/mg protein; Figure 5) since CS is a common biomarker used for mitochondrial density in skeletal muscle. Silva-Brito et al. found CS basal activities in juvenile seabass gills lower (10–17 nmol/min/mg protein) than those in liver (25–50 nmol/min/mg protein) [74]. In this study, the mycotoxin exposure did not affect the aerobic potential of the cells in gills (*p* > 0.05). However, a higher metabolic response was observed over time (between T0 and T28) in this tissue (*p* < 0.05), suggesting that some condition provided by the assay (ex. the increased fish movement due to shared environment and competition for food) could be influencing in some way. On the other hand, ENNB alone seems to have modified the cell capacity for aerobic metabolism in muscle because the CS activity was significantly inhibited in fish muscle (36%) exposed to this emerging mycotoxin (Figure 5). Probably, this reduction may be compensated with anaerobic pathways in order to sustain energetic demands of ENNB exposed fish, whereby lactate dehydrogenase (LDH) could confirm this hypothesis, since it is an important enzyme of the anaerobic metabolic pathway [75]. Since CS is commonly used as a quantitative enzyme biomarker for mitochondrial integrity, the observed reduction in CS activity indicates disturbances in metabolic pathways and functional modifications of mitochondrial capabilities. This loss of CS, along with decreased citrate levels, may hinder the cell’s ability to grow or proliferate in response to extracellular growth signals [76]. This most notorious effect of ENNB is evidenced in the retarded RGR observed in fish exposed to this compound via feed (Table 1). This lower aerobic capacity can also affect the mobility of the organism, making it slower [77]. Thus, the limited growth seems to be a strategy adopted to enhance the tolerance and survival under ENNB contamination. Moreover, the mycotoxin mixture as well as FB1 alone did not affect the aerobic capacity of gilthead seabream muscle.

## 3. Material and Methods

### 3.1. Experimental Design

The trial was performed using *Sparus aurata* juveniles reared at the aquaculture pilot station of the Portuguese Institute for the Sea and Atmosphere (EPPO-IPMA, Olhão, Portugal). Fish with an average weight of 65.4 ± 2.1 g were transported to IPMA’s Live Marine Organisms Bioterium (LabVivos, Algés, Portugal) and kept under optimal development conditions in recirculation aquaculture systems (RAS). Physico-chemical parameters were measured daily and adjusted, when necessary, in order to ensure the fish welfare, i.e., the water temperature was maintained at 19 °C, the dissolved oxygen around 7 mg/L, the pH at 8.0, the salinity at 35‰ and a photoperiod of 12 h light and 12 h dark (12L:12D). Fish were subjected to a quarantine period of 15 days during which the fish were fed a commercial (control) aquafeed, and subsequently distributed in experimental glass tanks of about 80 L (100 × 33 × 25 cm), where each treatment comprised three tanks (*n* = 6 per tank; *n* = 18 per treatment). The acclimation period lasted an additional 7 days prior to the exposure trial, during which the fish were also fed a commercial (control) aquafeed.

Upon acclimation, five treatments were carried out (*n* = 3 tanks/treatment; *n* = 6 fish per replicate tank; *n* = 18 per treatment in total): (i) control (CTRL); (ii) control with the solvent, i.e., absolute ethanol (CTRL + SOLV); (iii) fumonisin B1 (FB1); (iv) enniatin B (ENNB); and (v) a mixture of FB1 and ENNB (FB1 + ENNB). All treatments involved a mycotoxins concentration of around 150 µg/kg of dry weight (dw). The level tested was based on the levels estimated in 97 fish feeds considering a mean contamination scenario [7]. Before feeding, the feces and remnants of food were removed daily from the tanks by siphoning. Throughout the trial, fish were fed twice a day, totaling 1.7% of their body weight (bw) and a daily feed intake around 1 g/fish (i.e., chronic mycotoxins exposure occurred at a dose of 2.25 µg/kg bw per day).

### 3.2. Feed

Control and contaminated diets were manufactured by extrusion at SPAROS, Lda feed company (Olhão, Portugal) according to the nutritional requirements for juvenile gilthead seabream (a detailed formulation and the chemical composition are available in Supplementary Tables S1 and S2). Five different feeds were produced (CTRL, CTRL + SOLV, ENNB, FB1, and ENNB + FB1). Standards of ENNB (Sigma-Aldrich, E5411, West Chester, PA, USA) and FB1 (fumonisin B1 from *Fusarium moniliforme*; Sigma-Aldrich, F1147, West Chester, PA, USA) were added in the oil during the production process. The CTRL + SOLV treatment consisted of aquafeed without added mycotoxin, but with the equivalent amount of absolute ethanol used in the solubilization of mycotoxins. This treatment was performed in order to ensure that no carrier solvent toxicity occurred, because it would be expected that the low volume of the solvent was completely evaporated during feed production. Furthermore, no significant differences were found between the two control treatments (*p* > 0.05), i.e., CTRL with and without solvent, and therefore the CTRL with solvent was selected for comparisons with the three contaminated treatments (henceforth denominated as CTRL). The quality control analysis of the formulated feeds were performed to ensure the stability of the mycotoxins in the feeds. A total of three aliquots from each fish feed were collected, grinded in a domestic electric mincer (stainless steel, Becken) and analyzed (1 g) following the method described in Section 3.5 of the methodology. The resulting extract was analyzed twice in the LC-MSMS system.

To ensure the stability of the mycotoxin throughout the exposure period, all fish feeds were stored under dry conditions in the dark and analyzed before and during the assay to confirm real concentrations (FB1—139.05 µg/kg dw; ENNB—154.06 µg/kg dw; FB1 + ENNB—121.12 µg/kg dw and 130.10 µg/kg dw; CTRL and CTRL + SOLV—no detected).

### 3.3. Sample Collection

At the beginning (T0) and after 28 days of trial (T28), fish were randomly collected from each treatment to determine morphometric body condition indices (*n* = 6 per replicate tank and/or *n* = 18 in total per treatment) and to assess toxicological biomarkers (*n* = 6 per treatment). Upon 24 h of fasting (to allow for the total evacuation of feces before bulk-weighing) fish were euthanized by immersion for 5 min in an overdosed solution of the anesthetic tricaine methanesulfonate solution (MS-222; 2000 mg/L of, Sigma-Aldrich, USA) buffered with sodium bicarbonate (NaHCO_3_, Sigma-Aldrich, USA) using a ratio of 1:2 to adjust the pH and reduce fish stress. Immediately after euthanasia, a fraction of peripheral fish blood was collected with a syringe coated with heparin (3000 U/mL in saline solution 0.9% NaCl, Sigma-Aldrich, USA) by puncture of the caudal vein. Afterwards, the blood samples were centrifuged for 10,000× *g* at 4 °C during 10 min (Fisher Scientific AccuSpin Micro 17R Centrifuge, Osterode am Harz, Germany) to extract the supernatant (plasma; *n* = 2 per tank and/or *n* = 6 in total per treatment) that was immediately used for the determination of metabolites. Fish were subsequently dissected and samples of muscle, liver, brain, and gills were collected (*n* = 6 per treatment and for each tissue) and stored at −40 °C until further analyzes.

### 3.4. Fitness Parameters and Growth Performance

Morphometric measures (weight, length, and tissues weight) were obtained to evaluate the fish condition (*n* = 18 per treatment). The growth performance was assessed through relative growth rate (RGR, (1)) [78], while fitness was assessed through indices of animal condition according to [79], such as Fulton’s condition index (K) (2), and viscerosomatic (3) and hepatosomatic (4) indexes (VSI and HSI, respectively). The daily feed intake (DFI) (5) and the feed conversion rate (FCR) (6) were also calculated in dry weight through the formulas applied by Rodde et al. [80]. The uneaten feed weight was calculated every day and was considered in the DFI and FCR calculations.

(1) $$RGR\;(\%)=\frac{FW-IW}{IW}\;\times \;100$$

Here, FW is the final weight and IW is the initial weight (g, wet mass).

(2) $$K=\frac{TW}{{TL}^{3}}\;\times \;100$$

Here, TW is the total weight (g, wet mass) and TL is the total length of the fish (cm).

(3) $$VSI\;\left(\%\right)=\frac{VW}{TW}\;\times \;100$$

(4) $$HSI\;\left(\%\right)=\frac{LW}{TW}\;\times \;100$$

Here, VW is the fish viscera weight and LW is the fish liver weight (g, wet mass).

(5) $$DFI\;(g/fish)=\frac{feed\;intake\;per\;day\;(g)}{number\;of\;fish\;in\;the\;tank}$$

(6) $$FCR=\frac{total\;FI\;(g)}{weight\;gain\;(g)}$$

### 3.5. Mycotoxin Determination

Mycotoxin extraction in feed was performed with a modified QuEChERS method previously developed [81]. The contamination level of FB1 and ENNB in all feeds/treatments was systematically quantified during the experiment, using a MS/MS analysis that was performed on a Quattro Premier triple quadrupole mass spectrometer (Waters, Manchester, UK) interfaced with high-performance liquid chromatography (HPLC) system Waters Alliance 2695 (Waters, Milford, NH, USA). Seawater from each replicate tank was also collected and, in all samples, any transfer of both mycotoxins (either FB1 or ENNB) from aquafeeds to the seawater was not observed, since parent compounds were not detected. Mycotoxin extraction in water samples was performed with a modified extraction method according to Tolosa et al. [82], while in the other matrices (muscle, liver, gills, and brain) it was performed with a modified QuEChERS method that was previously developed [83].

Method accuracy and precision were evaluated using recovery studies. Precision was expressed as a percentage relative standard deviation (%RSD) of intra-day and inter-day repeatability. These parameters were determined with replicate samples (*n* = 3) spiked at three concentration levels (25, 200, and 400 µg/kg). The limit of quantification (LOQ) was defined as the lowest concentration of the analyte that could be quantified with acceptable precision (<20%) and accuracy (>70% and 120%). The limit of detection (LOD) was determined with a 3:1 signal-to-noise ratio. Linearity was evaluated with matrix-matched calibration curves (25–800 µg/kg).

### 3.6. Plasma Biochemical Analysis

The fish plasma biochemical profile was assessed using a compact veterinary chemistry analyzer (PT10V Samsung Electronics, Suwon, Republic of Korea). Thirteen analyzes (alanine aminotransferase [ALT], albumin [ALB], alkaline phosphatase [ALP], amylase [AMY], blood urea nitrogen [BUN], calcium [Ca], creatinine [CREA], total cholesterol [CHOL], gamma-glutamyl transpeptidase [GGT], globulin [GLOB], glucose [GLU], lipase [LIPA], phosphorus [PHOS], total bilirubin [TBIL], and total protein [TP]) and two ratios (albumin/globulin [A/G] and blood urea nitrogen/creatinine [B/C]) were quantitatively determined in 70 µL of fish plasma (*n* = 6 per treatment) by spectrophotometry after sample reaction in a specific cartridge. Quality control was assured with results in compliance with the specified cut-off values for each control (QC material).

### 3.7. Biomarker Analysis

**Figure 6:**
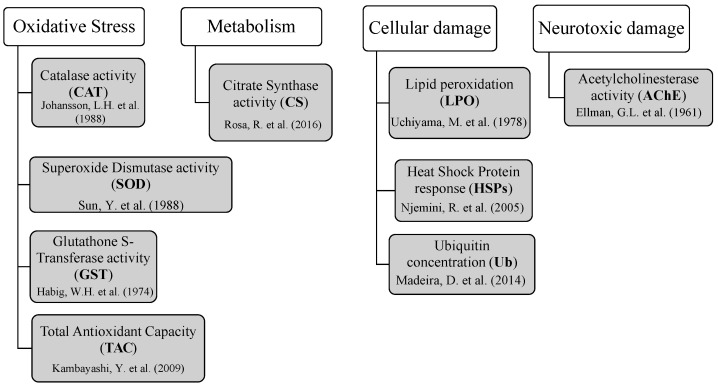
Biomarkers of oxidative stress, aerobic metabolism, and cellular and neurotoxic damage assessed to investigate toxicological responses in gilthead seabream exposed to mycotoxins through aquafeed [84,85,86,87,88,89,90,91,92].

Nine toxicological biomarkers were analyzed to assess fish tissue response to mycotoxin exposure (*n* = 6 per treatment; Figure 6). Activities of catalase (CAT), superoxide dismutase (SOD), and glutathione S-transferase (GST) were determined by spectrophotometric enzymatic assays adapted [84,85,86], respectively. Antioxidant defenses were investigated in fish muscle, brain, liver, and gills. However, gills samples were not analyzed for SOD since the limited amount of this tissue was not enough to perform all the analyzes. Total antioxidant capacity (TAC) was measured using an ABTS decolorization assay as described by Kambayashi et al. [87]. This method involves a spectrophotometric assessment of Trolox that is analogous to vitamin E, a natural antioxidant. As an indicator of aerobic potential, citrate synthase activity (CS) was measured in muscle and gills by spectrophotometric enzymatic assays based in Rosa et al. [88].

Lipid peroxidation (LPO) was measured in all studied tissues as the total malondialdehyde (MDA) content through the thiobarbituric acid test, adapted from Uchiyama and Mihara [89]. Protein chaperoning was determined through heat shock response (HSPs), assessed by an enzyme-linked immunosorbent assay (ELISA) that quantifies the HSP70/HSC70 content, based on the methodology described by Njemini et al. [90]. For this biomarker, the brain was not assessed due to the limited amount of samples. In addition, the ubiquitin content (Ub) was determined in all tissues through the ELISA methodology, as described by Madeira et al. [91], to identify protein degradation and/or DNA repair. The detection ranges of HSP70 and ubiquitin were 0.156–2 μg/mL and 0.002–1 μg/mL, respectively. Intra-inter-assay coefficients of variation were below 10% for HSP70 and below 15% for Ub, indicating a good repeatability and reproducibility of the measurements.

Finally, acetylcholinesterase activity (AChE) was measured in muscle and brain by a spectrophotometric enzymatic assay adapted from Ellman et al. [92] to investigate neurotoxicity effects. All spectrophotometric readings were performed in a spectrophotometer microplate reader (Thermo Scientific, model Multiskan GO 1510, Waltham, MA, USA with Skanit software 3.2). Each sample was analyzed in duplicate and read twice.

The total protein content was also quantified in each sample according to the Bradford assay [93], in order to normalize the results of each biomarker, which were expressed in U/mg of protein, except for SOD (% of inhibition). Reagents of a pro analysis or higher grade were used for all biomarker analyzes as well as 96-well microplates from Nunc (Roskilde, Denmark).

### 3.8. Statistical Analysis

Prism 6 for Windows (GraphPad software, Inc., CA, USA) was used for statistical and graphic analysis and a statistical significance was set at a level of *p* < 0.05. All data were expressed as mean ± standard deviation (mean ± SD).

Regarding data from biomarkers and the plasma biochemical profile, firstly, the assumptions for the parametric tests were assessed using the Shapiro–Wilk test for normality and the Brown–Forsythe test for homogeneity of variances. After checking these assumptions, a One-Way ANOVA test was applied followed by Tukey’s HSD post-hoc test to detect significant differences between experimental treatments for each fish tissue. When data did not comply with these assumptions, data were transformed (log or square-root). However, when these transformations did not sufficiently meet the assumptions for parametric analysis, non-parametric tests were applied. Specifically, the Kruskal–Wallis test followed by the Dunn’s post-doc test for multiple comparisons were performed to detect significant differences among the treatments. For pairwise comparisons between the two sampling times (T0 and T28), either the Student’s *t*-test (for parametric data) or the Mann–Whitney U test (for non-parametric data) was used.

## 4. Conclusions

In conclusion, the current study clearly shows that the exposure to ENNB and FB1 (whether alone or in combination) at levels around 150 μg/kg lead to distinct effects on different fish tissues, with varied responses in biochemical biomarkers after 28 days of exposure. These differences were likely influenced by the distinct functioning, physiology, and baseline levels of the tissues. Nonetheless, exposure to ENNB alone was the most harmful to juvenile *S. aurata*, causing protein and lipid degradation in liver, and affecting lipase and alkaline phosphatase activities in plasma.

This study also highlights the importance of including emerging ENNB in the current legislation for fish feeding, particularly for fish, to ensure the well-being and health of farmed fish and consequently human food safety. More in-depth research involving other mycotoxins (particularly non-regulated), in isolation or combination, as well as the exposure to higher levels of contamination in shorter time periods are strongly encouraged. This would improve our understanding of the possible synergistic, antagonistic, or additive effects in commercial aquatic species. Moreover, it would contribute to the understanding of the toxicological responses observed, whether they are time-, concentration- or species-specific, and the metabolization timing of these compounds in fish.

## Figures and Tables

**Table 1:** The mean values ± standard deviation (ww, *n* = 12 per treatment) of morphometric measures (total weight and total length), animal fitness (K, VSI, and HSI), growth performance (RGR), and feed efficiency (FI and FCR) of juvenile *S. aurata* exposed to four treatments at day 0 (T0) and after 28 days (T28).

	Weight (g)	Total Length (cm)	K	VSI	HSI	RGR (%)	FI (g/fish/day)	FCR (g feed/g)
T0	65.1 ± 5.0	16.4 ± 0.8	1.5 ± 0.1	5.2 ± 0.5	2.3 ± 0.2	-	-	-
CTRL (T28)	75.3 ± 7.7 b *	16.8 ± 0.4 b	1.5 ± 0.1 b	5.1 ± 0.3	2.6 ± 0.3 a *	25.8 ± 6.9 a	1.06	1.44 a
ENNB (T28)	79.4 ± 4.6 b	17.1 ± 0.2 b	1.6 ± 0.5 b	5.2 ± 0.4	2.5 ± 0.2 a	13.4 ± 3.2 b	1.13	2.74 b
FB1 (T28)	90.3 ± 6.0 a	17.8 ± 0.5 a	1.6 ± 0.1 b	4.8 ± 0.3	2.0 ± 0.2 b	24.3 ± 1.2 a	1.11	1.26 a
FB1 + ENNB (T28)	87.8 ± 5.1 a	17.1 ± 0.6 b	1.7 ± 0.1 a	5.2 ± 0.4	2.5 ± 0.3 a	29.0 ± 1.9 a	1.14	1.46 a

Asterisks (*) represent significant differences (*p* < 0.05) between sampling times T0 and T28 (CTRL; *t*-test). Different letters (a,b) indicate significant differences (*p* < 0.05; ANOVA) among the four treatments at T28. CTRL—control; FB1—fumonisin B1; ENNB—enniatin B; K—Fulton’s condition factor; VSI—viscerosomatic index; HSI—hepatosomatic index; RGR—relative growth rate; FI—daily feed intake; FCR—feed conversion rate.

**Table 2:** Plasma biochemical analytes for juvenile gilthead seabream (*S. aurata; n* = 6 per treatment) after a 28-day exposure to mycotoxins.

	GLU (mg/dL)	BUN (mg/dL)	CREA (mg/dL)	B/C	PHOS (mg/dL)	Ca (mg/dL)	TP (g/dL)	ALB (g/dL)	GLOB (g/dL)	A/G	ALT (U/L)	ALP (U/L)	CHOL (mg/dL)	LIPA (U/L)	AMY (U/L)
CTRL	85.6 ± 23.8	10.6 ± 1.8	0.20 ± 0.1	58.5 ± 17.9	9.7 ± 1.1	12.4 ± 0.4	4.0 ± 0.3	1.3 ± 0.1	2.7 ± 0.3	0.48 ± 0.1	35.2 ± 9.5	339 ± 84	348.2 ± 44	24.4 ± 3.3	22.6 ± 4.3
FB1	90.6 ± 21.4	11.8 ± 3.4	0.20 ± 0.1	57.2 ± 38.7	9.0 ± 0.2	12.3 ± 0.6	3.9 ± 0.3	1.2 ± 0.0	2.7 ± 0.3	0.46 ± 0.1	27.6 ± 3.6	261.2 ± 35	316.8 ± 91	24 ± 5.3	20.8 ± 5.5
ENNB	86.6 ± 8.0	7.9 ± 1.7	0.38 ± 0.2	40.3 ± 11.7	10.5 ± 1.3	12.7 ± 1.2	4.4 ± 0.2	1.3 ± 0.1	2.8 ± 0.7	0.42 ± 0.0	51.2 ± 29.2	229 ± 40 *	302.4 ± 92	36.5 ± 9.7 *	29.8 ± 18.8
FB1 + ENNB	95.8 ± 9.41	7.8 ± 1.5	0.18 ± 0.1	57.6 ± 34.7	9.4 ± 1.1	12.2 ± 0.4	3.9 ± 0.3	1.3 ± 0.1	2.7 ± 0.2	0.46 ± 0.1	31.4 ± 2.6	312 + 56	334.2 ± 58	27.7 ± 4.7	21.4 ± 6.4

Asterisks (*) indicate a statistically significant difference compared to the control treatment (CTRL, *p* < 0.05). Abbreviations: ALT—Alanine aminotransferase; ALB—albumin; ALP—alkaline phosphatase; AMY—amylase; A/G—albumin/globulin ratio; B/C—blood urea nitrogen/creatinine ratio; BUN—blood urea nitrogen; Ca—calcium; CREA—creatinine; CHOL—total cholesterol; GLOB—globulin; GLU—glucose, LIPA—lipase; PHOS—phosphorus; TP—total protein. CTRL—control; FB1—fumonisin B1; ENNB—enniatin B.

## Data Availability

The original contributions presented in this study are included in the article/Supplementary Material. Further inquiries can be directed to the corresponding authors.

## Supplementary Materials

The following supporting information can be downloaded at https://www.mdpi.com/article/10.3390/ijms26125676/s1.
