# Supplementary material for: Toxicological Responses of Juvenile Gilthead Seabream to Enniatin B and Fumonisin B1
Source: Int J Mol Sci. 2025 Jun 13;26(12):5676. doi: 10.3390/ijms26125676 (PMC12193222; doi:10.3390/ijms26125676)
Supplement: Supplementary file 1 [file ijms-26-05676-s001.zip › ijms-3622285-supplementary.pdf]

SUPPLEMENTARY MATERIAL

Table S1. Detailed composition of ingredients in the commercial aquafeed used for the experiment with gilthead seabream. Pellets had 3 mm.

Ingredients	%
Fishmeal Super Prime	17.00
Krill meal	3.00
Poultry meal	12.00
Soy protein concentrate	10.00
Wheat gluten	7.50
Corn gluten meal	10.00
Soybean meal Hipro (Alphasoy 530)	10.00
Wheat meal	14.00
Vitamin and mineral premix	1.00
Choline chloride 50%	0.20
Antioxidant	0.20
Monoammonium phosphate	0.80
Chromic oxide	1.00
Fish oil	4.00
Algae oil	1.30
Rapeseed oil	8.00

Table S2. Nutritional and chemical composition of the commercial aquafeed used for gilthead seabream (3 mm).

Composition	
Crude protein, % feed	47.1
Digestible protein, %	41.7
Crude fat, % feed	16.0
Crude fat (no coating)	16.0
Fiber, % feed	1.3
Starch, % feed	12.0
Ash, % feed	8.1
Gross energy, MJ/kg feed	21.0
Digestible energy, MJ/kg feed	17.7
DP/DE	23.5
Arg, % feed	2.6
His, % feed	1.0
Ile, % feed	1.8
Leu, % feed	3.9
Lys, % feed	2.4
Thr, % feed	1.7
Trp, % feed	0.5
Val, % feed	2.1
Met, % feed	0.9
Cys, % feed	0.6
Met + Cys, % feed	1.6
Phe, % feed	2.2
Tyr, % feed	1.6
Phe + Tyr, % feed	3.7
Asx, % feed	3.8
Glx, % feed	9.0
Ala, % feed	2.6
Gly, % feed	2.8
Pro, % feed	3.3
Ser, % feed	2.2
Tau, % feed	0.1
Total P, % feed	1.1
Phytic acid, %	0.3
Phytate P, % feed	0.1
Digestible P, %	0.73
Ca, % feed	1.2
Ca/P	1.0
Na, % feed	0.3
Mg, % feed	0.2
K, % feed	0.7
Cu, mg/kg feed	13.4
Fe, mg/kg feed	159.2
I, mg/kg feed	3.7
Mn, mg/kg feed	28.6
Se, mg/kg feed	0.9

Zn, mg/kg feed	113.1
Vit A, IU/kg feed	28581.8
Vit D3, IU/kg feed	2257.2
Vit K3, mg/kg feed	30.9
Vit E, mg/kg feed	624.2
Vit B1, mg/kg feed	37.7
Vit B2, mg/kg feed	37.6
Vit B3, mg/kg feed	525.3
Vit B5, mg/kg feed	126.9
Vit B6, mg/kg feed	38.9
Vit B9, mg/kg feed	18.8
Vit B12, mg/kg feed	0.3
Vit C, mg/kg feed	619.9
Biotin, mg/kg feed	2.0
Choline, mg/kg feed	1604.0
Inositol, mg/kg feed	618.9
Betaine, mg/kg feed	1414.4
Astaxanthin,mg/kg feed	0.0
Canthaxanthin, mg/kg feed	0.0
Apo-ester, mg/kg feed	0.0
Beta-carotene, mg/kg feed	0.0
C14, % feed	0.2
C16, % feed	2.0
C18, % feed	0.3
C18:1n9, % feed	5.0
LNA (C18:2n6), % feed	2.0
ALA (C18:3n3), % feed	0.7
ARA, % feed	0.1
EPA, % feed	1.1
DHA, % feed	2.4
EPA+DHA, % feed	3.51
Total phospholipids, % feed	0.3
